# Mechanism of nitrogen metabolism-related parameters and enzyme activities in the pathophysiology of autism

**DOI:** 10.1186/1866-1955-4-4

**Published:** 2012-02-13

**Authors:** Ghada A Abu Shmais, Laila Y Al-Ayadhi, Abeer M Al-Dbass, Afaf K El-Ansary

**Affiliations:** 1 Biochemistry Department, Science College, King Saud University, P.O box 22452, Zip code 11495, Riyadh, Saudi Arabia; 2 Department of Physiology, Faculty of Medicine, King Saud University, Riyadh, Saudi Arabia; 3 Autism Research and Treatment Center, Riyadh, Saudi Arabia; 4 Shaik AL-Amodi Autism Research Chair, King Saud University, Riyadh, Saudi Arabia

## Abstract

**Background:**

There is evidence that impaired metabolism play an important role in the etiology of many neuropsychiatric disorders. Although this has not been investigated to date, several recent studies proposed that nitrogen metabolism-related parameters may have a pathophysiological role in autism.

**Methods:**

The study enrolled 20 Saudi boys with autism aged 4 to 12 years and 20 healthy controls matched for age and gender. Levels of creatine, urea, ammonia, gamma-aminobutyric acid (GABA), glutamate:glutamine (Glu:Gln) ratio, and enzymatic activities of glutamate dehydrogenase, 5'-nucleotidase, and adenosine deaminase (ADA) were determined in plasma samples from both groups.

**Results:**

We found a significant elevation of creatine, 5'-nucleotidase, GABA, and glutamic acid and a significant decrease in the enzymatic activity of ADA and glutamine level in patients with autism compared with healthy controls. The most significant variation between the two groups was found in the Glu:Gln ratio.

**Conclusion:**

A raised Glu:Gln ratio together with positive correlations in creatine, GABA, and 5'-nucleotidase levels could contribute to the pathophysiology of autism, and might be useful diagnostic markers. The mechanism through which these parameters might be related to autism is discussed in detail.

## Background

Autism is a complex disorder that is heterogeneous in nature, with varying degrees of severity, and for which no specific biological marker has been identified. A recent epidemiological study in Saudi Arabia established the autism prevalence at 6:1000 (Talat; unpublished data, personal communication). The increasing prevalence of autism is raising public-health concerns [1]. As indicated by several biological parameters, a hallmark of autism is its substantial heterogeneity. Although the core deficits may fall in the similar domains, no two people with autism have exactly the same profile. Such a wide range of clinical features makes the diagnosis and treatment of autism very challenging. The overlapping of different biological and genetic etiologies and the individual variations in patients with autism makes it difficult to establish unified diagnostic biomarkers and therapeutic strategies [2,3].

Autism involves many metabolic derangements, such as those involving the trans-sulfation pathway that synthesizes cysteine, glutathione, sulfate, and taurine. Abnormalities also include an imbalance between antioxidation systems and production of reactive oxygen species, which results in disruption of cellular molecules and subsequent damage. In addition, a role for mild mitochondrial dysfunction in autism has been suggested [4-6].

Nitrogen metabolism and nitrogen compounds are important and essential components in the life-cycle of living organisms, The simplest useful form of nitrogen is ammonia, which only a few organisms can synthesize from atmospheric nitrogen. Therefore, amino groups are carefully husbanded in nature. Nitrogen greatly contributes to the mass and composition of biological systems, with most of it being in the form of amino acids. During metabolic turnover of proteins and nucleic acids, nitrogen-containing molecules are often salvaged and reused [7].

Unlike other tissues, the brain lacks two urea cycle enzymes, carbamoyl phosphate synthetase (CPS)-1 and ornithine transcarbamylase (OTC), and is therefore unable to remove nitrogen as urea. The main route for ammonia disposal in the brain is via glutamine synthesis from glutamate by glutamate synthase (GS) in astrocytes. In neurons, glutamine can be deaminated to glutamate by glutaminase. The generated ammonia then escapes through the extracellular space into astrocytes to participate in the GS reaction. This creates a gln-glu cycle between neurons and astrocytes [8]. Another important but minor route for disposal of ammonia is amination of α-ketoglutarate (α-KG) to glutamate by glutamate dehydrogenase (GDH) [7]. In addition to their crucial energetic roles in the CNS, ATP and adenosine are important in neurotransmission. Adenosine is a neuromodulator of brain function that is uniquely positioned to integrate excitatory and inhibitory neurotransmission [9]. Adenosine metabolism in the brain is very important, and its dysregulation has been implicated in several neurological disorders. Intracellular and extracellular levels of adenosine are tightly controlled by specific nucleoside transporters and several important enzymes, which include adenosine deaminase (ADA) and 5'-nucleotidase (5'-NT) [10-12].

Nitric oxide (NO) is a neurotransmitter and/or neuromodulator in both the central and peripheral nervous systems. In the CNS, it is associated with pain perception and control of sleep, appetite, thermoregulation, learning and memory, neural development, and synaptic plasticity. Depending on its concentration, NO exerts a biphasic effect on the release of several neurotransmitters and neuromodulators, including glutamate, gamma-aminobutyric acid (GABA), serotonin (5-HT), with low concentrations decreasing their release and high concentrations increasing release [13].

The crucial role of creatine (Cr) and phosphorylcreatine (PCr) as components of the CK system to maintain energy requirements is well established. However, Cr is thought to have additional important functions in the CNS [14]. For example, recent findings suggest a neuroprotective role of Cr against ammonia toxicity, preventing impairment of axonal growth [15]. Cr is also involved in regulation of glycolysis, stabilization of mitochondrial creatine kinase (mtCK), and inhibition of mitochondrial permeability transition pore (MPTP) opening, an early event in apoptosis [16].

Based on these findings, we aimed in the present study to measure a number of selected biochemical parameters related to nitrogen metabolism in the plasma of patients with autism from Saudi Arabia, and compared these with the levels in healthy controls matched for age and gender.

## Methods

### Ethics approval

The study protocol followed the ethical guidelines of the most recent Declaration of Helsinki [17]. Written informed consent provided by the parents of all subjects enrolled in the study according to the guidelines of the ethical committee of King Khalid Hospital, King Saud University, and assent to participate was obtained from the subjects themselves if they were developmentally able.

### Subjects

In total, 20 patients with autism (18 males 2 females, mean age ± standard deviation (SD) 8 ± 4 years, range 4 to 12 years) were enrolled through the Autism Research and Treatment (ART) Center clinic from a sample population consisting of children diagnosed with autism spectrum disorder (ASD). The diagnosis of ASD was confirmed in all subjects using the Autism Diagnostic Interview-Revised (ADI-R) criteria [18], the Autism Diagnostic Observation Schedule-Generic (ADOS-G) criteria [19], and the Developmental, Dimensional and Diagnostic Interview (3DI) [20]. All patients had autism simplex, and all were negative for Fragile X. No patients were on special diets or alternative treatments.

The control group was recruited from the Well Baby clinic at King Khaled University Hospital (all male 16 males 4 females, mean age ± SD 7.5 ± 3.5 years, range 4 to 11 years). All participants were screened via parental interview for current and past physical illness.

Exclusion criteria included presence of organic aciduria, dysmorphic features, a diagnosis of Fragile X or other serious neurological (for example, seizures), or psychiatric (for example, bipolar disorder) conditions, or known medical conditions, including endocrine, cardiovascular, pulmonary, liver, kidney or other medical disease.

### Blood samples

After an overnight fast, patients underwent blood sampling; 10 ml blood samples were collected on ice from both groups in test tubes containing heparin as anticoagulant. The samples were separated by centrifugation at *3000 rpm *and 25°C for 10 minutes. The plasma was removed and frozen at -80°C until analyzed.

### Biochemical analyses

#### Assay of 5'-nucleotidase (5'-NT) and adenosine deaminase (ADA) enzyme activities

5'-NT and ADA were assayed using commercial diagnostic kits (BioQuant Inc., San Diego, CA, USA). The assays are based on the enzymatic hydrolysis of 5'-inosinemonophosphate (5'-IMP) or the enzymatic deamination of adenosine by 5'-NT or ADA, respectively to form inosine, which is converted to hypoxanthine by purine nucleoside phosphorylase (PNP). Hypoxanthine is then converted to uric acid and hydrogen peroxide (H_2_O_2_) by xanthine oxidase (XOD). H_2_O_2 _is further reacted with N-ethyl N-(2-hydroxy-3-sulfopropyl)-3-methylaniline (EHSPT) and 4-aminoantipyrine (4-AA) in the presence of peroxidase (POD) to generate a quinone dye, which was monitored using a spectrophotometer set to the kinetic mode for 3 min, at 550 nm, with 1 min interval [21,22].

#### Measurement of glutamate dehydrogenase activity

For measurement of glutamate dehydrogenase activity, a commercial kit (Randox Laboratories Ltd., Crumlin, Co. Antrim, UK) was used. This is an optimized standard method according to the recommendations of the Deutsche Gesellschaft für Klinische Chemie. The substrates α-xoglutarate and ammonium are converted to glutamate and water by GDH in the sample, using NADH as coenzyme. This procedure measures the non-specific creep reaction through the change in NAD(H) absorbance at 340 nm [23].

#### Measurement of ammonia and urea

Ammonia and urea were measured using diagnostic kits (Randox Laboratories). The kits work in a similar way. The ammonia in the sample combines with α-oxoglutarate and NADH in the presence of GDH to yield glutamate and NAD^+^. The corresponding decrease in absorbance at 340 nm is proportional to the plasma ammonia concentration [24]. Similarly, for urea, the enzyme urease converts water and the urea in the sample to ammonia and carbon dioxide. Salicylate and hypochlorite in the reagent react with the ammonium ions to form a green complex (2,2-dicarboxylindophenol), which is measured at 600 nm [25].

#### Measurement of creatine

The creatine assay used is an accurate, convenient measure of creatine for a variety of biological samples (BioVision Inc., Milpitas, CA, USA). In the assay, creatine is enzymatically converted to sarcosine, which is then specifically oxidized to generate a product that converts a colorless probe to an intensely red color (λ_max _= 570 nm), and a strongly fluorescent (Ex/Em = 538/587 nm) product. Creatine is therefore easily detected by either colorimetric or fluorometric methods. The detection range is 0.001 to 10 mmol/l creatine [26].

#### Measurement of nitric oxide

A colorimetric assay was used, which provides an accurate, convenient measure of total nitrate/nitrite in a simple two-step process (Biovision). The first step is the conversion of nitrate to nitrite by nitrate reductase. The reagents react with nitrite only, and not nitrate. The second step uses Griess reagents to convert nitrite to a deep purple azo compound, and the amount of the azo chromophore accurately reflects nitric oxide amount in samples. The detection limit of the assay is approximately 0.1 nmol nitrite/well, or 1 μmol/l [27].

#### Assay of GABA

Quantitative determination of GABA was performed using ELISA immunoassay kit (ALPCO Diagnostics, Salem, NH, USA). An aliquot (300 μL) of each sample type (diluted standard, control or undiluted samples) was placed in each well, then 300 μl of the diluent was added. The plate was covered with adhesive foil and agitated on a shaker at 600 rpm for 30 minutes at room temperature (20 to 25°C). Two washing cycles were performed, through incubation with 1 ml of I-buffer for 5 min at RT (20-25°C) on a shaker, after which 250 μl of elution buffer was placed into the appropriate wells of the extraction plate, which was covered and placed on the shaker for 10 minutes. Next, 100 μl of each extract was used for derivatization. NaOH (10 μl) was added into each well, followed by 50 μl of the equalizing reagent (freshly prepared before each assay) and the plates shaken for 1 minute at 600 rpm. Then, 10 μl of the D-reagent was added into each well, and the plate incubated for 2 hours at room temperature on a shaker., after which 150 μl of the buffer supplied with the kit (Q-buffer) was added into each well, and the plate incubated for 10 min at room temperature at 600 rpm. From each well, 25 μl was removed and transferred to the GABA microtiter strips to start the ELISA. To that, 50 μl of GABA antiserum was added, mixed, covered, and incubated for 2 hours at RT on a shaker. After 3 washings with 300 μl wash buffer, 100 μl of enzyme conjugate was added to the dry wells, covered, and incubated for 30 min again at RT with shaking. After another 3-washings and drying step, 100 μl of substrate (TMB) was added with incubation for 20-30 min at RT on a shaker. Finally, 100 μl of the stop solution was added with shaking, and the absorbance was read within 10 minutes, using a microplate reader set to 450 nm and a reference wavelength between 620 nm and 650 nm.

#### Measurement of glutamate and glutamine levels

Glutamate and glutamine levels were assessed using an HPLC method [28]. Plasma samples (0.1 ml) were mixed with 5 μl mercaptoethanol and allowed to stand for 5 minutes at room temperature, then precipitated with ice-cold methanol while being vortexed. Tubes were allowed to stand for 15 minutes in an ice bucket before samples were separated by centrifugation (5000 rpm for 15 minutes) and the supernatant was collected. The efficiency of the protein precipitation step was assessed by Bradford's dye-binding method [29]. The protein-free supernatants were processed immediately for HPLC analysis of the two amino acids.

### Statistical analyses

Statistical Program for Social Sciences (SPSS) (SPSS Inc., Chicago, IL, USA) was used for all analyses. Results were expressed as mean ± SD. All statistical comparisons were made by means of Student's *t*-test. *P *< 0.05 was considered significant. The data was also illustrated using normal distribution, mean, and percentage for each parameter and group. Pearson's correlation coefficient was used to find a correlation between the measured parameters. Generally, positive or negative correlations above 0.80 are considered very high. The receiver operating characteristic (ROC) curve, a fundamental tool for biomarker evaluation, was performed using the same computer program. In a ROC curve, the true positive rate (sensitivity) is plotted in function of the false positive rate (100-specificity) for different cut-off points of a parameter. Each point on the ROC curve represents a sensitivity/specificity pair corresponding to a particular decision threshold. The area under the ROC curve is a measure of how well a parameter can distinguish between patients with autism and control subjects.

## Results

**Figure 1:**
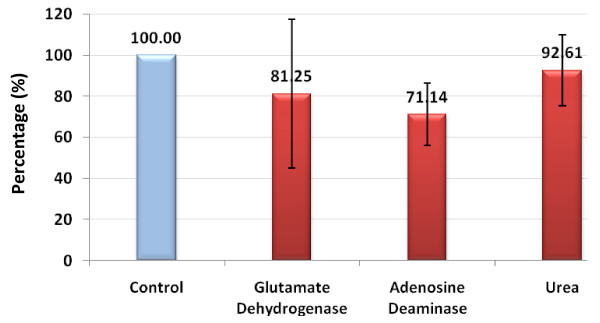
**Percentage decrease in glutamate dehydrogenase (GDH), adenosine deaminase (ADA) and urea in patients with autism compared with controls**.

**Figure 2:**
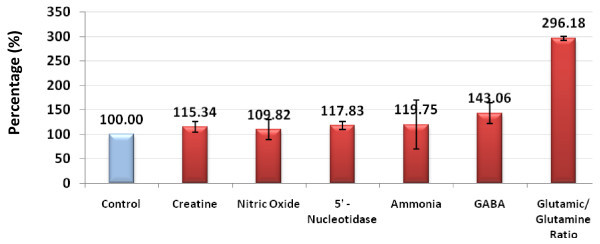
**Percentage increase in creatine (Cr), nitric oxide (NO), 5'-nucleotidase (5'-NT), ammonia (NH_3_), gamma-aminobutyric acid (GABA), and glutamate:glutamine (Glu:Gln) ratio in patients with autism compared with controls**.

**Figure 3:**
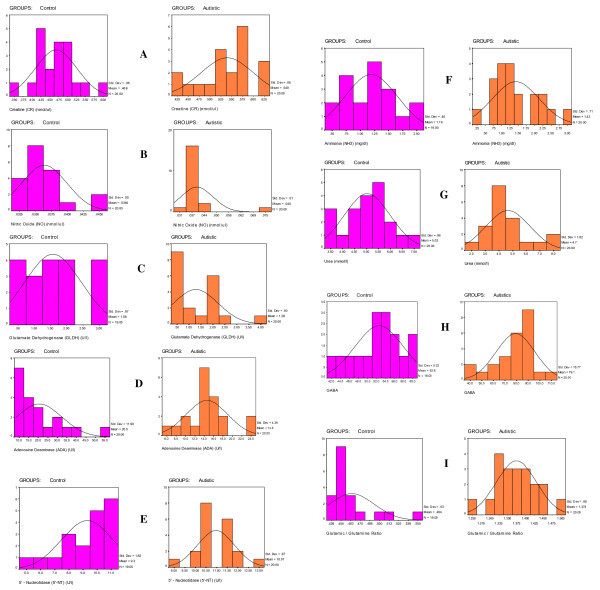
**Normal distribution for the various measured parameters in the control and autistic groups**. The scale of the X axis is different for the two groups, because of the marked differences between them.

We compared the levels of the various chemicals and the activities of some enzymes in the plasma of autistic and control groups (Table 1; Figure 1; Figure 2; Figure 3).

**Table 1:** Mean ± SD of the measured chemicals in plasma of patients with autism compared with age-matched controls.^a^

	Group	Mean ± SD	*P *value
Cr, nmol/ul	Control	0.47 ± 0.06	0.000
	Autistic	0.54 ± 0.06	
NO, nmol/ul	Control	0.036 ± 0.00	0.084
	Autistic	0.040 ± 0.01	
GDH, U/l	Control	1.58 ± 0.87	0.311
	Autistic	1.28 ± 0.93	
ADA, U/l	Control	20.47 ± 11.90	0.048
	Autistic	14.56 ± 4.39	
5'-NT, U/l	Control	9.31 ± 1.82	0.001
	Autistic	10.97 ± 0.87	
NH_3_, mg/dl	Control	1.19 ± 0.46	0.230
	Autistic	1.43 ± 0.71	
Urea, mmol/l	Control	5.03 ± 0.96	0.382
	Autistic	4.66 ± 1.62	
GABA, ng/ml	Control	55.29 ± 4.15	0.023
	Autistic	79.09 ± 16.77	
Glu, μmol/l	Control	111.9 ± 4.63	0.001
	Autistic	152.8 ± 16.77	
Gln, μmol/l	Control	241.82 ± 13.29	0.001
	Autistic	111.34 ± 5.69	
Glu:Gln	Control	0.46 ± 0.03	0.001
	Autistic	1.37 ± 0.06	

^a^Abbreviations: 5'-NT, 5'-nucleotidase; ADA, adenosine deaminase; Cr, creatine; GABA, gamma-aminobutyric acid; GDH, glutamate dehydrogenase; Gln, glutamine; Glu, glutamate; NH_3_, ammonia; NO, nitric oxide.

Of these, only creatine, ADA and 5'-NT were significantly different between the autistic group and the control group (Table 1, Figure 1, Figure 2). The autistic group had an increase in nitric oxide and ammonia of 9.82% and 19.75%, respectively, compared with the control group, but this was non-significant. There was also a decrease in levels of GDH and urea of 18.75% and 8.31% respectively, in the autistic group compared with the control group, but again, this was non-significant.

There was a marked increase in both GABA and Glu:Gln levels in patients with autism compared with controls (Table 1; Figure 2; Figure 3H, I] Of 25 patients with autism, 22 had GABA levels of 60 ng/ml or higher, whereas only 5 of 16 controls had levels of 54 ng/ml or higher. Overall, there was a 43% increase in GABA in patients with autism compared with controls (Figure 2). There was a marked increase in the Glu:Gln ratio, with all (20/20; 100%) of patients with autism having ratios greater than 1.25, whereas all the controls had ratios of less than 0.55; overall, there was an increase of 196.18% increase in the Glu:Gln ratio in patients with autism compared with controls (Figure 2).

**Figure 4:**
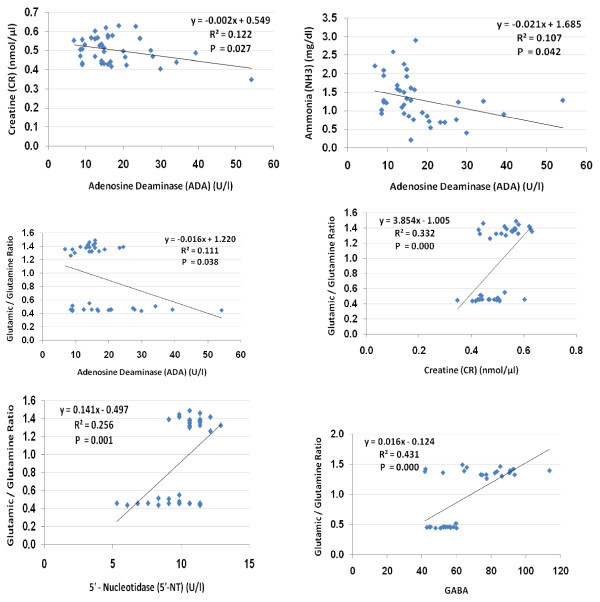
**Pearson correlations between the measured parameters showing the best-fit line curve with either positive or negative correlations**.

When Pearson correlation coefficients were calculated, it was apparent that ADA was negatively correlated with Cr and NH_3 _(*P *< 0.03 and *P *< 0.05, respectively),. The Glu:Gln ratio was positively correlated with creatine, 5'-NT and GABA, and negatively correlated with ADA only (Table 2; Figure 4)

**Table 2:** Pearson correlation (*R*) between the measured parameters^a^

Parameters	*R *	*P *value
ADA and Cr	-0.351^b^	0.027

ADA and NH_3_	-0.327^b^	0.042

ADA and Glu:Gln	-0.334^b^	0.038

Glu:Gln and Cr	0.576^a^	0.000

Glu:Gln and 5'-NT	0.506^a^	0.001

Glu:Gln and GABA	0.657^a^	0.000

^a^Abbreviations: 5'-NT, 5'-nucleotidase; ADA, adenosine deaminase; Cr, creatine; GABA, gamma-aminobutyric acid; Gln, glutamine; Glu, glutamate; NH_3_, ammonia.

^b^Negative correlation.

^c^Positive correlation.

**Figure 5:**
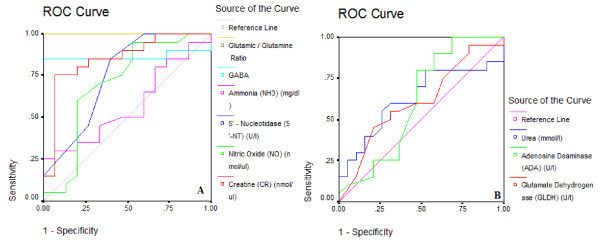
**Receiver operating characteristic (ROC) curve of **(A) **creatine (Cr), nitric oxide (NO), 5'-nucleotidase (5'-NT), ammonia (NH_3_), gamma-aminobutyric acid (GABA), and glutamate:glutamine (Glu:Gln) ratio and **(B) **of glutamate dehydrogenase (GDH), adenosine deaminase (ADA), and urea in autistic group**.

Using ROC analysis, the highest values for specificity and sensitivity were found for the Glu:Gln ratio, GABA, 5'-NT and Cr (Table 3; Figure 5A, B).

**Table 3:** Receiver operating characteristic analysis of the measured parameters showing area under the curve (AUC), specificity and sensitivity of each.

Parameter	AUC	Best cut-off value	Sensitivity, %	Specificity, %
Cr	0.812	0.511	75.0	85.0

NO	0.770	0.036	95.0	55.0

GDH	0.608	1.084	55.0	68.0

ADA	0.624	16.226	80.0	55.0

5'-NT	0.787	10.247	85.0	68.0

NH_3_	0.586	2.107	25.0	100.0

Urea	0.637	5.196	80.0	50.0

GABA	0.866	61.74	85.0	100.0

Glu:Gln	1.000	0.906	100.0	100.0

^a^Abbreviations: 5'-NT, 5'-nucleotidase; ADA, adenosine deaminase; Cr, creatine; GABA, gamma-aminobutyric acid; GDH, glutamate dehydrogenase; Gln, glutamine; Glu, glutamate; NH_3_, ammonia; NO, nitric oxide.

## Discussion

Despite the fact that neurological disorders can be caused by many different primary defects, they often produce similar impairments in cellular energy metabolism in the brain. In these instances, intracellular concentration of ATP is decreased, resulting in cytosolic accumulation of Ca^2+ ^and generation of reactive oxygen species (ROS). Ca^2+ ^and ROS, in turn, trigger apoptotic or necrotic cell death. For many of these disorders, impairments of brain Cr metabolism have also been described, including decreases in total Cr concentration, PCr concentration, CK activity, and/or creatine transporters (CrT) content [30-32]. Similarly, knockout mice lacking the brain cytosolic and mitochondrial isoenzymes of CK displayed a slightly increased Cr concentration, but no PCr in the brain. These mice had decreased weight gain and reduced life expectancy, disturbed fat metabolism, behavioral abnormalities, and impaired learning capacity [32].

In this study, we found that patients with autism from Saudi Arabia had raised levels of plasma Cr compared with age-matched controls (Table 1; Figure 2; Figure 3). Increased plasma Cr could be due to defective Cr transport to the brain, a suggestion supported by recent work by Kara *et al. *[33], in which they showed that cerebral Cr deficiency (as detected *in vivo *by magnetic resonance spectroscopy (MRS) of the brain) and specific disturbances in metabolites of Cr metabolism in body fluids was linked to mental retardation, expressive speech and language delay, epilepsy, developmental delay, and autistic behavior [33]. In addition, autistic features were seen in patients with guanidinoacetate methyltransferase (GAMT) deficiency as an inborn error of Cr biosynthesis. The diagnosis of GAMT deficiency is based on excessive amounts of guanidinoacetate in body fluids, and decreased levels of Cr/PCr in the brain [34,35]. Our speculated decrease of brain Cr occurring concomitantly with raised plasma Cr in Saudi patients with autism is also in line with the work of Battini *et al. *[36] in which ^1^H-MRS disclosed brain Cr depletion in 2-year-old child who presented with psychomotor and language delay, and autistic-like behavior.

NO has been recognized as a biological neural messenger molecule, although it is best known as a toxic reactive free radical in the CNS. NO or NO-derived nitrogen oxides must interact with and modify neuromodulators, especially in patients with autism compared with controls, which indicates a possible role of nitric oxide synthase in the pathogenesis of autism [37]. Increased oxidant end-products produced by the reactions of NO with other free radicals may possibly contribute to the psychopathology of autism because of the preferential vulnerability of the brain of patients with autism to oxidative injury, as shown in a previous Saudi study [5]. Although we did not find a significant increase in NO in the Saudi boys with autism in the present study, the level was almost 10% higher than that in controls, thus there is still a suggestion that NO might contribute to the pathogenesis of autism. The lack of significance might be attributable to the finding in a recent study by Al-Yafee *et al. *who reported a significant increase in peroxidoxins, which are very efficient scavengers of NO [38].

ADA (EC 3.5.4.4) is an enzyme catalyzing hydrolytic deamination of either adenosine or deoxyadenosine to inosine and deoxyinosine, respectively. Because of the irreversibility of the reaction catalyzed by ADA, this enzyme reaction seems to be one of the rate-limiting steps in adenosine degradation [39]. Detoxification of adenosine and deoxyadenosine is important because they are toxic to cells in high concentrations. Several mechanisms for this toxicity have been proposed. One possible explanation is that high concentrations of adenosine and deoxyadenosine cause dATP accumulation in the cell; dATP is a strong inhibitor of ribonucleotide reductase and causes some aberrations in DNA synthesis [40].

Adenosine mediates its actions through the activation of specific G-protein-coupled receptors (adenosine receptors; ARs), of which four subtypes have been identified (A1R, A2AR, A2BR and A3R) [41]. These receptors have distinctive pharmacological profiles, tissue distributions and effector-coupling mechanisms, and their functioning has been extensively studied in the CNS [42]. Interestingly, A1R and A2AR are largely responsible for the central effects of adenosine [43]. A2AR is mostly coupled to G-proteins in the peripheral systems, and mediates its effects predominantly through activation of adenylyl cyclase, which in turn converts ATP into cAMP. It is well known that low concentrations of adenosine activate predominantly A1R, which inhibits glutamate release. High concentrations of adenosine activate A2AR, which, by means of the A1R/A2AR intramembrane interaction, antagonizes A1R function, therefore facilitating glutamate release [44]. Moreover, the modulatory effects of adenosine on dopamine systems have been investigated, in view of their relevance to human disorders such as schizophrenia and Parkinson's disease. Antagonistic adenosine-dopamine interactions have been widely reported, showing that adenosine can inhibit several effects of dopamine in the cerebral cortex and basal ganglia [45]. Therefore, a possible relationship between the losses of adenosine homeostasis due to the currently reported reduced activity of ADA, and the impairment of neurotransmitter profile is suggested in patients with autism. This is also supported by the findings of our most recent work in which we reported low plasma and high brain serotonin and dopamine concentration in patients with autism compared with control subjects [46].

Based on these results, it seems likely that tight control of adenosine levels could play an important role in brain development and neural plasticity [47], and that any dysfunction in homeostatic control of adenosine, an important modulator of the brain immune system, could upset the balance between pro-inflammatory and anti-inflammatory cytokines, which is crucial for normal brain development [48]. Together, these findings support a contribution of the decreased activity of ADA reported in the present study to the dysfunction in normal adenosine homeostasis in Saudi patients with autism during prenatal and perinatal brain development. The decreased ADA activity recorded in the present study is in good agreement with an earlier study by Stubbs *et al. *who reported reduced activity of ADA in subjects with autism compared with that in healthy controls, patients with mental retardation, and patients with cerebral palsy [49]. Lower ADA activity in patients with autism might also be related to the increased levels of interleukin-6 and tumor necrosis factor-α, and the impaired Ca^2+ ^and K^+ ^homeostasis that were reported from two studies using the same investigated autistic samples [50,51].

It is well known that 5'-NT activity is increased in response to abnormal accumulation of some nucleotide substrates [52]. In the present study, the significant increase in 5'-NT activity in patients with autism compared with control subjects might be related to the significantly lower ADA activity recorded in the same samples, as lower ADA could lead to the accumulation of adenosine and AMP. This suggestion is supported by previous work from Page *et al. *[53], who identified four unrelated patients in whom developmental delay, seizures, ataxia, recurrent infections, speech deficit, and an unusual autistic behavioral phenotype were associated with increased activity of 5'-NT and more recent work by Page [54] showing that 5'-NT superactivity is one of the most important metabolic defects associated with autistic symptoms.

We found that GABA was significantly higher in the plasma of Saudi patients with autism, with a 43% increase compared with controls (Table 1; Figure 2). This is in good agreement with the previous work of Dhossche and Rout [55], who reported that children with autism have increased plasma GABA levels. This increase may also be associated with reduced cerebral GABA, which might be the result of a reduced number of neurons expressing this neurotransmitter [56,57]. This suggestion is supported by the work of Hollander *et al. *in which they reported that inhibition of the catabolic enzymes of GABA using divalproex was effective in treating boys with autism [58]. The patients were described as having affective instability (for example, they were impulsive and aggressive) and retrospective analysis suggested that 10 of the patients (72%) had improved behavior after divalproex treatment, which might be due to increases in GABA in the brain.

One distinctive strategy that serves to accomplish the marked stratification of brain glutamate levels is the Glu-Gln cycle. To minimize the risk of excitotoxicity, the intrasynaptic glutamate must be kept at a very low level. At the same time, neurons require a steady supply of precursors to replenish the glutamate lost via oxidative processes. The Glu-Gln cycle in the brain serves to control the levels of glutamate, and to shuttle nitrogen between astrocytes and neurons. Under basal conditions, glutamate is metabolized in astrocytes by the GS reaction rather than by GDH, favoring glutamine formation [59,60]. In the present study, the significant increase in the Glu:Gln ratio that we found in patients with autism compared with controls suggest that the Glu-Gln cycle was greatly affected in these patients. This could be related to the increased levels of ammonia and the marked reduction in urea concentration reported in the present study.

## Conclusion

**Figure 6:**
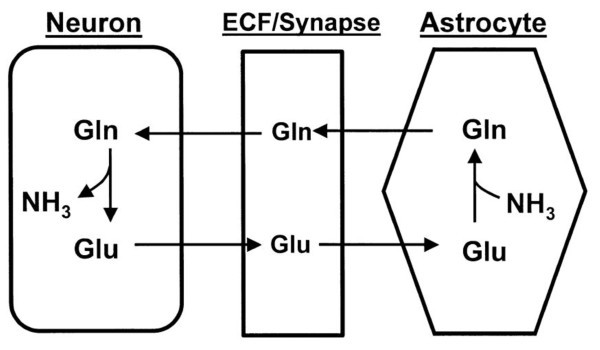
**The Glu-Gn cycle in the brain **[**59**].

**Figure 7:**
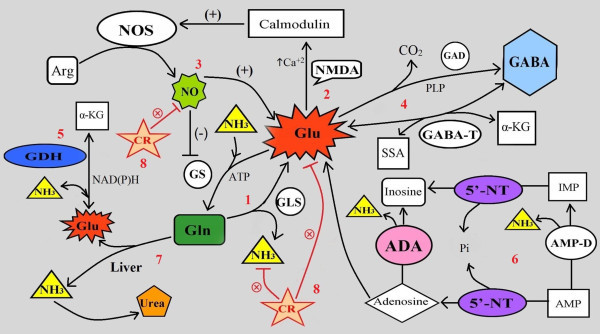
**Interactions between key metabolites and pathways suggested by the results of the present study as potential diagnostic biomarkers of autism**.

The use of potential biomarkers that indicate specific mechanisms of disordered neurodevelopment should help to identify meaningful subtypes of autism and tailor treatment or prevention strategies. A number of the parameters we investigated had decreased or increased levels in the patients with autism compared with the age-matched control subjects. Glutamate excitotoxicity is probably the final common pathway or mechanism through which impaired nitrogen metabolism might be involved in the etiology of autism. The increased Glu:Gln ratio and the positively correlated abnormal levels of Cr, GABA, and 5'-NT might be useful diagnostic markers for autism (Figure 6). We have outlined the relationship of glutamate and glutamine to most of the measured parameters (Figure 7), and illustrated the interactions between the key metabolites and pathways that we suggest might play a role in the pathophysiology of autism.

## List of abbreviations

4-AA: 4-aminoantipyrine; 5-HT: serotonin; 5'-IMP: 5'-inosine monophosphate; 5'-NT: 5'-nucleotidase; α-KG: α-ketoglutaric acid; ADA: adenosine deaminase; CNS: central nervous system; CPS-1: carbamoyl phosphate synthetase-1; Cr: creatine; EHSPT: N-ethyl N-(2-hydroxy-3-sulfopropyl)-3-methylaniline; GABA: gamma-aminobutyric acid; GDH: glutamate dehydrogenase; Gln: glutamine; Glu: glutamate; GS: glutamate synthase; H_2_O_2_: hydrogen peroxide; HPLC: high-performance liquid chromatography; mtCK: mitochondrial creatine kinase; MPTP: mitochondrial permeability transition pore; NO: nitric oxide; OTC: ornithine transcarbamylase; PCr; phosphorylcreatine; PNP: by purine nucleoside phosphorylase; POD: peroxidase; XOD: xanthine oxidase.

## Competing interests

The authors declare that they have no competing interests.

## Authors' contributions

GAA carried out the biochemical assays. LYA confirmed the diagnosis, provided the samples, and obtained ethics approval. AMA participated in performing the statistical analysis. AKE designed the study and drafted the manuscript. All authors have read and approved the final manuscript.
